# Applications of *In Vivo* Imaging in the Evaluation of the Pathophysiology of Viral and Bacterial Infections and in Development of Countermeasures to BSL3/4 Pathogens

**DOI:** 10.1007/s11307-014-0759-7

**Published:** 2014-07-10

**Authors:** Thomas M. Bocan, Rekha G. Panchal, Sina Bavari

**Affiliations:** 1 Molecular and Translational Sciences, US Army Medical Research Institute of Infectious Diseases (USAMRIID), 1425 Porter Street, Ft. Detrick, MD 21702 USA; 2 The Geneva Foundation, 917 Pacific Ave, Suite 600, Tacoma, WA 98402 USA

**Keywords:** *In vivo* imaging, MRI, PET, SPECT, CT, Optical, Ultrasound, BSL3/4 pathogens

## Abstract

While preclinical and clinical imaging have been applied to drug discovery/development and characterization of disease pathology, few examples exist where imaging has been used to evaluate infectious agents or countermeasures to biosafety level (BSL)3/4 threat agents. Viruses engineered with reporter constructs, *i.e.*, enzymes and receptors, which are amenable to detection by positron emission tomography (PET), single photon emission tomography (SPECT), or magnetic resonance imaging (MRI) have been used to evaluate the biodistribution of viruses containing specific therapeutic or gene transfer payloads. Bioluminescence and nuclear approaches involving engineered reporters, direct labeling of bacteria with radiotracers, or tracking bacteria through their constitutively expressed thymidine kinase have been utilized to characterize viral and bacterial pathogens post-infection. Most PET, SPECT, CT, or MRI approaches have focused on evaluating host responses to the pathogens such as inflammation, brain neurochemistry, and structural changes and on assessing the biodistribution of radiolabeled drugs. Imaging has the potential when applied preclinically to the development of countermeasures against BSL3/4 threat agents to address the following: (1) presence, biodistribution, and time course of infection in the presence or absence of drug; (2) binding of the therapeutic to the target; and (3) expression of a pharmacologic effect either related to drug mechanism, efficacy, or safety. Preclinical imaging could potentially provide real-time dynamic tools to characterize the pathogen and animal model and for developing countermeasures under the U.S. FDA Animal Rule provision with high confidence of success and clinical benefit.

## Introduction

Preclinical and clinical *in vivo* imaging approaches have been widely utilized in the characterization of disease and drug efficacy across numerous therapeutic areas, most notably, neuroscience, oncology, cardiovascular, and immunology. Few examples exist where *in vivo* imaging has been applied to the evaluation of infectious agents, anti-infective drug discovery, and/or biosafety level (BSL)3/4 biothreat agents. There are very likely several practical reasons for the limited use of *in vivo* imaging in the assessment of BSL3/4 infectious agents such as the need for significant biocontainment facilities of which there are few where imaging capabilities exist, isolation of imaging hardware from infected animals, and the rapid onset of the disease in infected animals. Attempts to image animals infected with BSL3 agents have employed self-contained isolation chambers, *i.e.*, sealed tubes [1, 2] to avoid contamination. Some groups have installed imaging systems within a BSL2 environment to allow for easier hardware maintenance or utilized large Philips Bioshield^™^ polycarbonate plastic tubes extending from the BSL4 containment space into the bores of the various scanners to isolate the hardware from the infectious agents [3]. Others have installed the imaging equipment directly into BSL3 containment and developed procedures for infected animal isolation and hardware decontamination, *e.g.*, gaseous paraformaldehyde or hydrogen peroxide. All of the above approaches come with various disadvantages such as the requirement for modified imaging hardware to accommodate the containment barriers, reduced image resolution, scatter and attenuation, reduced flexibility or higher cost of operation with regard to using disposable components, *e.g.*, magnetic resonance imaging (MRI) surface coils, and the potential exists for a reduced hardware lifespan due to exposure to caustic decontamination solvents. Animal isolation approaches and imaging outside of containment limit the breadth of pathogens that could be utilized due to safety concerns related to isolation chamber malfunction, security risks associated with taking infected animals outside containment, and risks of exposure due to personnel errors.

Despite the challenges, *in vivo* imaging could play a significant role in better understanding the pathophysiology of infectious agents and in the discovery and development of therapies for BSL3/4 pathogens. Given the nature of the pathogens and despite the absence of drugs to treat the infections, classical methods of drug development cannot be applied. The development pathway for anti-viral and anti-bacterial products against viral and bacterial threat agents is complex because clinical efficacy studies may not be feasible or ethical. In these instances, the U.S. FDA Animal Rule allows animal efficacy data to be used along with human safety evaluation data and pharmacokinetic information to support drug approval [4]. Under the Animal Rule, a thorough understanding of the pathophysiology of the agent in the animal is needed so as to qualify that the model used for drug efficacy assessment and for linkage to phase I human pharmacokinetic/pharmacodynamic parameters is representative of the human condition.

*In vivo* imaging is well suited to provide a dynamic assessment of pathogen infection, disease progression, and resolution following drug intervention. The *in vivo* imaging tools are non-invasive except when using contrast agents, *e.g.*, MRI, computed tomography (CT), and ultrasound (US), or semi-invasive, *e.g.*, positron emission tomography (PET), single photon emission computed tomography (SPECT), and optical, and when used together can serially monitor both structural and functional changes associated with disease progression. In this article, we will review (1) our basic understanding of viral and bacterial infections with an emphasis on BSL3 and BSL4 agents; (2) the general principles underlying the various imaging modalities; (3) application of preclinical *in vivo* imaging in drug discovery; and (4) the current approaches where *in vivo* imaging has been applied to evaluate viral and bacterial pathogens and drug/countermeasure interventions.

## Processes Involved in Viral and Bacterial Infections

Viral and bacterial infections involve a series of steps that while not independent are pathogen and route of inoculation specific such as cellular uptake, replication and spread of the pathogen, and host specific such as modulation of the innate and adaptive immune response to the infectious agent. To identify the steps where *in vivo* imaging can play a role in better understanding the pathogenesis of the infectious agents, we will briefly review the life cycle of a viral agent, Venezuelan equine encephalitis virus (VEEV), and bacteria agent, *Burkholderia pseudomallei*, which are potential biothreat agents.

**Fig. 1:**
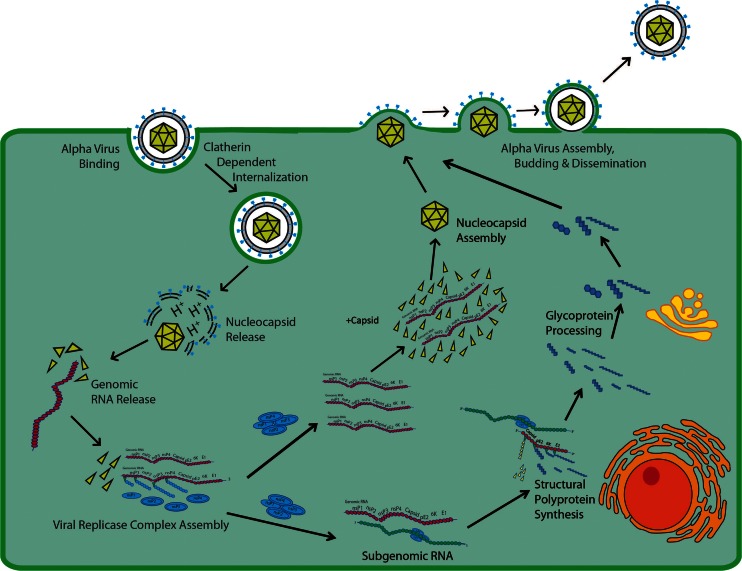
Diagrammatic representation of the process of alphavirus infection.

Alphaviruses are small, *i.e.*, 65–70 nm, encapsulated spherical particles containing a positive-strand genomic RNA of approximately 11.5 kb in length. Alphaviruses referred to as “old world” viruses such as Sindbis virus (SINV), Semliki Forest virus, Ross River virus (RRV), and Chikungunya (CHIKV) cause rheumatic diseases in humans [5]. The “new world” viruses, Venezuelan, Eastern, and Western equine encephalitis virus (VEEV, EEEV, WEEV), cause fatal encephalitic diseases in the Americas [6]. The general process of alphavirus infection (for review, see references [7–10]) is depicted in Fig. 1. The alphavirus binds to cell receptors mediated by the viral E2 glycoprotein and internalized in a clathrin-dependent manner. Under low pH, the nucleocapsid is released into the cytoplasm through a fusion pore of viral and endosomal membranes where it disassembles to expose the RNA for translation in cytoplasmic vacuoles. The viral replicase complex assembles upon translation of the non-structural proteins with minus-strand RNA synthesis occurring early and both plus-strand and subgenomic RNA synthesis occurring late in infection. Genomic RNA is transcribed while structural proteins involved in capsid formation are translated from subgenomic RNA as a polyprotein of capsid-pE2-6K-E1. The polyprotein inserts into the endoplasmic reticulum for further processing while the genomic RNA and capsid are assembled in the cytoplasm. Fusion of the newly synthesized nucleocapsid and viral glycoproteins occurs at the cell membrane and budding of new virus results. To mitigate an immune response, alphavirus infection shuts down the host transcription and translation processes without affecting virus replication, decreases IFN-α/β production which reduces the innate immune system and host anti-viral responses, and promotes cytopathic responses responsible for induction of the apoptotic pathway. In the cases of alphavirus-induced encephalitis and articular disease/myalgia, the hallmark host response is macrophage infiltration, cytokine and chemokine release, and edema.

**Fig. 2:**
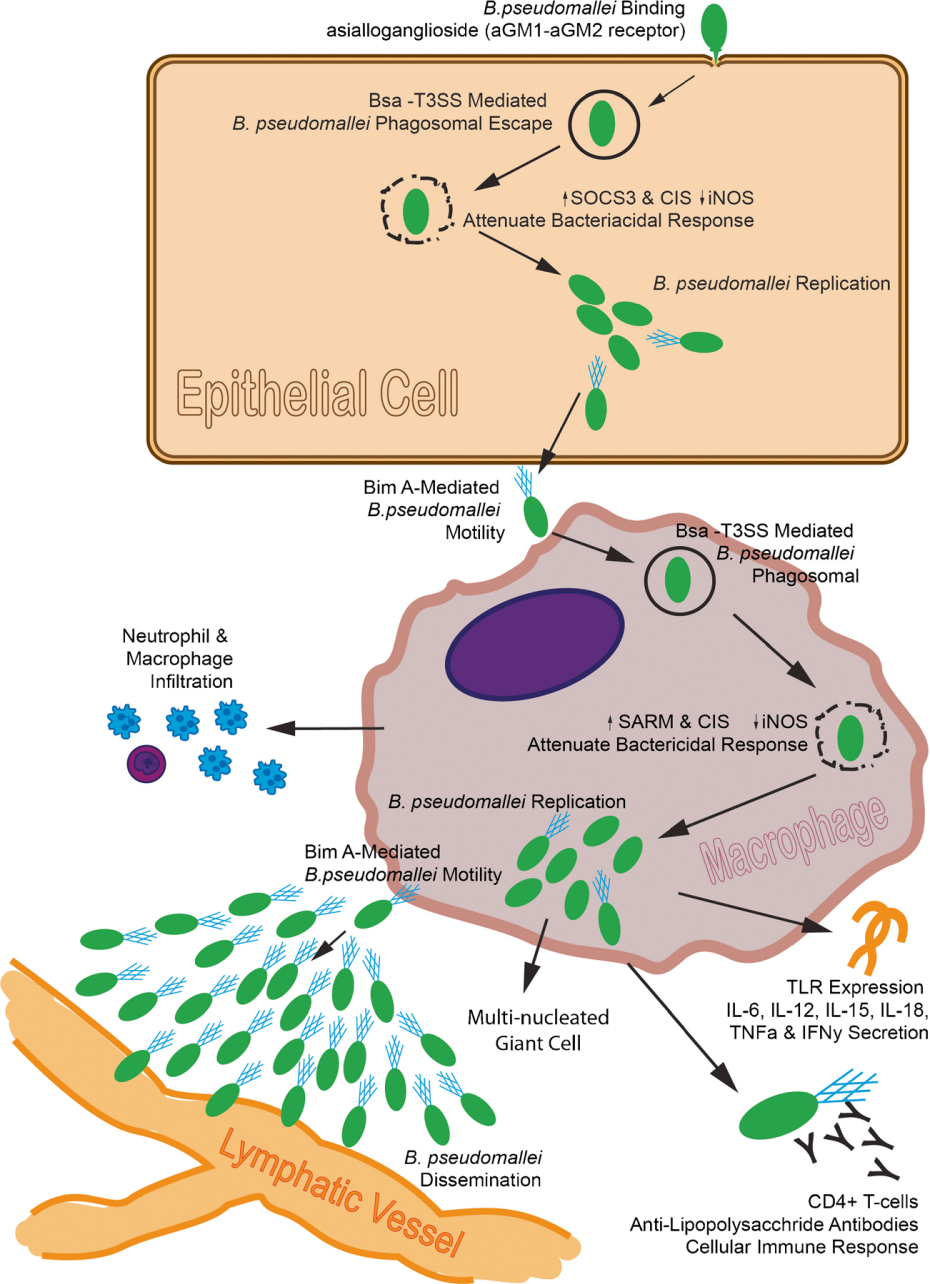
Diagrammatic representation of *Burkholderia pseudomallei* infection.

*B. pseudomallei* is a gram-negative bacteria measuring 2–5 μm in length and 0.5–0.8 μm in diameter and which is endemic to tropical areas in Southeast Asia and Northern Australia. Infection with *B. pseudomallei* can occur *via* percutaneous inoculation, inhalation, or aspiration, and the sensitivity to infection and resulting melioidosis is dependent on the individual’s immune status and presence of underlying conditions such as diabetes, renal disease, and alcohol abuse. The process of *B. pseudomallei* infection (see reference [11, 12] for review) is diagrammatically represented in Fig. 2. Following inhalation exposure, *B. pseudomallei* binds to the pharyngeal epithelial cell presumably through an asialoganglioside aGM1–aGM2 receptor complex mediated by the bacterial type IVA pili. The method of invasion of the bacteria into the cell is unknown, but the event is associated with rearrangement of the host actin cytoskeleton induced by the *Burkholderia* secretion apparatus (Bsa) type 3 secretion system (T3SS). In epithelial cells, *B. pseudomallei* represses inducible nitric oxide synthase (iNOS) by activating expression of a suppressor of cytokine signaling 3 (SOCS3) and cytokine-inducible src homology 2-containing protein (CIS). Upon phagocytic or non-phagocytic cellular uptake, the bacteria appear in vacuoles and with the aid of T3SS escapes into the cytoplasm where it replicates. In macrophages, replication continues without activating a bactericidal response. Repression of the bactericidal response is associated with a reduction in reactive oxygen or nitrogen intermediates. In macrophages, *B. pseudomallei* represses iNOS and interferon-β expression by activating sterile-α and Armadillo motif (SARM) containing protein [13]. Bacterial spread is accomplished through macrophage lysis and through intracellular spread by membrane protrusions to nearby cells or by cell fusion to produce multi-nucleated giant cells. *B. pseudomallei* travel between cells by actin-mediated motility involving BimA to form actin tails. Dissemination of the bacteria within the host is likely accomplished through macrophages or transport through the lymphatic system within a polysaccharide and lipopolysaccharide capsule which protects the bacteria from complement-mediated killing and provide resistance to cationic peptides, respectively [11]. The acute host response to *B. pseudomallei* is a rapid influx and activation of neutrophils followed by macrophage infiltration and stimulation of an immune response mediated through Toll-like receptors (TLRs) 2 and 4 plus expression of the proinflammatory cytokines IL-6, IL-10, IL-12, IL-15, IL-18, TNFα, and IFNγ which contribute to the tissue destruction and pathogenesis of melioidosis. The cellular immune response requires the presence of macrophages and CD4+ T cells, and anti-lipopolysaccharide antibodies appear to be a potential mechanism for bactericidal activity.

Host pathogen interactions are complex and involve a diverse range of mechanisms by which the pathogens can cause disease. However, the cellular process of infection typically involves common steps of binding, internalization, replication, muted host anti-viral and anti-bacterial response, budding, and distribution within the host to initiate disease. Disease initiation and progression, irrespective of alphavirus or *B. pseudomallei* infection, involves a pronounced inflammation of brain, joints, or lung resulting in encephalitis, arthralgia, or melioidosis, respectively. Beyond using optical and nuclear approaches to demonstrate the distribution of a modified pathogen containing a reporter construct, *in vivo* imaging could provide dynamic, serial information about disease progression following a viral and bacterial infection. Given the relative paucity of information utilizing imaging to study infectious agents, the subsequent sections will review applicable imaging tools used in the evaluation of disease processes of relevance to infectious agents, *e.g.*, inflammation and neurologic disorders. In addition, the uses of imaging in drug discovery will be highlighted and specific applications of where imaging has been used to study infectious agents will be summarized.

## Imaging in Drug Discovery and Development

### Basic Principles of Imaging

Over the course of the last decade, imaging devices such as optical (bioluminescent and fluorescent), PET, SPECT, CT, US, and MRI have been developed for use in preclinical studies. Comparison of the resolution, sensitivity, and key features of each imaging modality is summarized in Table 1. Optical imaging is a measure of light emitted from a probe, *e.g.*, green fluorescent protein and luciferase-luciferin, and used primarily in nude or non-pigmented mice, as a reporter of a specific cellular process or demonstration of the distribution of a target or cell. PET is a nuclear approach which detects two 511-keV photons emitted at 180° apart from an annihilation reaction of a positron produced from the decay of an unstable exogenously delivered tracer such as fluorine-18, carbon-11, and copper-64 coupled to a target molecule of interest, *e.g.*, 2-deoxy-2- [^18^F]fluoro-d-glucose ([^18^F]-FDG), which is used to measure a specific enzymatic, receptor, or protein interaction. When PET is coupled with CT or MRI, the radiotracer reagents can be co-localized to specific regions within organs with finer precision. SPECT imaging detects gamma-emitting isotopes, *e.g.*, technetium-99m (Tc-99m) and iodine-123 (I-123), conjugated to targets of interest much like PET, and some isotopes can be used to image molecules such as proteins and antibodies for longer periods of time. CT is a three-dimensional measure of X-ray attenuation properties of different tissues, and while lacking in innate contrast sensitivity, it provides very high spatial resolution, which is detector size and X-ray dose-dependent. CT provides high-resolution structural images of bone and lung and with the addition of contrast agents is somewhat capable of soft tissue imaging. Ultrasound is a two-dimensional measure of reflected high-frequency sound waves from a tissue of interest to create a structural image of the tissue in the transducer field-of-view and can be used in Doppler mode for perfusion with or without bubbles to improve contrast resolution. MRI is a three-dimensional measure of proton magnetization where image contrast is a function of the tissue environment within which the proton resides and can be interrogated with various techniques, *e.g.*, bladder—may appear bright on a T2 sequence but dark on a T1 sequence. MRI like CT has very high soft tissue contrast resolution without the need for ionizing radiation. Taken together, optical, PET, and SPECT while not exclusively are best characterized as modalities amenable to molecular and functional imaging and CT, US and MRI are best utilized for anatomical imaging; however, some protocols with and without contrast agents have been developed to measure functional processes such as blood flow.

**Table 1:** Comparison of the imaging modalities

Imaging modality	Resolution (μm)	Sensitivity	Key features
Optical	2,000	pM–fM	Two-dimensional Limited depth of penetration Molecular and functional imaging Mice (nude or non-pigmented) only
PET	1,500–2,000	nM–pM	Three-dimensional Short- and long-lived isotopes Molecular and functional imaging
SPECT	100–200^a^	nM–pM	Three-dimensional Potential for imaging multiple probes simultaneously Molecular and functional imaging
CT	30+	μM	Three-dimensional Primarily bone and lung imaging Soft tissue imaging requires contrast agents Anatomical imaging
Ultrasound	30+	μM	Two-dimensional Depth of penetration dependent on transducer frequency User dependent due to manual manipulation of transducers Anatomical imaging
MRI	10+	μM	Three-dimensional No ionizing radiation Anatomical imaging^b^

^a^Resolution is scanner dependent

^b^Potential for functional imaging using contrast agents

### Applications of Imaging in Drug Discovery

Coincident with the development of the preclinical imaging tools, their application to the drug discovery and development process has become more widespread. Imaging is well-suited to evaluate (1) the presence of a therapeutic target or drug at their specific site of action; (2) binding of the therapeutic to the target; and (3) expression of a pharmacologic effect either related to drug mechanism, efficacy, or safety. Applications of imaging for assessment of these three processes can be found in neuroscience, oncology, cardiovascular, and immunology. Examples, while not exhaustive with regard to what is available in the literature, are highlighted below to demonstrate how imaging has been applied to these disease areas to address the above three questions and to exemplify by extension how imaging can address processes of relevance to infectious diseases.

In neuroscience, the utilization of PET tracers has become a somewhat standard method for demonstrating brain receptor or enzyme expression and drug occupancy, while MRI is utilized to quantify structural and/or functional processes such as flow, perfusion, diffusion, and neuronal activation. There are numerous examples of the use of imaging in the study of neuroscience, neuropathology, and drug intervention [14–16]. Most recently, a novel phosphodiesterase 2A (PDE2A) tracer, 4-(3-[^18^F]fluoroazetidin-1-yl)-7-methyl-5-{1-methyl-5-[4-(trifluoromethyl)phenyl]-1H-pyrazol-4-yl}imidazo[5,1-f]-[1,2,4]triazine ([^18^F]PF-05270430), was identified through a rational PET tracer design methodology [17]. In cynomologus monkeys, [^18^F]PF-05270430 was used to demonstrate the distribution of PDE2A to brain striatum (putamen and caudate) and not cerebellum and while not shown such binding was noted to be blocked in a dose-dependent manner by a specific PDE2A inhibitor [17]. When the tissue distribution, drug binding to a specific target and PK data are taken together, one can correlate plasma drug levels with the specific target occupancy needed for drug efficacy. Novel PET tracers have been used to characterize the progression of disease such as Alzheimer’s by quantifying the deposition of beta-amyloid [18–20]. More general PET tracers such as [^18^F]-FDG can be used to discern changes in brain metabolism and drug effects related to both efficacy and safety. Magnetic resonance imaging and MR spectroscopy have been used to further characterize Alzheimer’s disease pathology by assessing changes in brain size [21] and neuroinflammation by measuring myoinositol levels [21, 22].

Application of imaging to the diagnosis and assessment of treatment in oncology has been long-standing. Multimodality approaches (for review, see [23]) have been used preclinically to characterize growth and metastasis of xenograft tumors and orthotopic tumors because the traditional caliper measurements inconsistently follow functional changes. Measures of tumor metabolism and proliferation have employed 2-deoxy-2-[^18^F]fluoro-d-glucose ([^18^F]FDG) and [^18^F]fluoro-3-dexoythymidine ([^18^F]FLT) PET, respectively. Staging the degree of tumor proliferation with [^18^F]FLT has provided more consistent cohorts of animals for drug testing, reduced variability in the measurement, and demonstrated a pharmacologic effect prior to tumor size changes [24, 25]. Dynamic contrast-enhanced MRI (dceMRI) is routinely used to characterize tumor angiogenesis and changes in blood flow following treatment [26]. From a preclinical perspective, optical imaging of tumors containing fluorescent or bioluminescent reporter constructs has enabled rapid compound testing and staging of tumor growth prior to applying the more complex PET, SPECT, or MRI approaches. In addition to assessing drug mechanism or efficacy, imaging has been used to phenotype tumors, *e.g.*, expression of carcinoembryonic antigen [27], to better define the appropriate course of clinical treatment and to select potentially more responsive patient cohorts.

Cardiovascular imaging has focused primarily in the areas of atherosclerosis and diabetes to characterize disease progression and disease-modifying therapies. [^18^F]FDG is used as a marker of vascular inflammation because macrophages resident within the atherosclerotic lesion exhibit an increased metabolic activity [28, 29]. Treatment with simvastatin reduces macrophage accumulation within human atherosclerotic plaques and decreases [^18^F]FDG activity consistent with such findings [30]. In diabetes, to quantify the mass of pancreatic β-islet cells, a novel tracer, [^11^C]dihydrotetrabenazine, which targets β-cell vesicular monoamine transporter type II (VMAT2) has been shown to detect pancreatic β-islet cells and reduction in cell mass in streptozotocin-treated and Zucker rat models of diabetes [31] and in human type I diabetics [32].

Immunological responses such as with inflammation are present across multiple disease areas, and applications for imaging can be found in rheumatoid arthritis [33] and atherosclerosis [28–30] as noted above and infection and general inflammation [34]. In general, [^18^F]FDG has been used substantially to assess inflammation. More selective markers for the peripheral-type benzodiazepine receptor (PBR) or what is also referred to as the 18-kDa translocator protein (TSPO), R-[^11^C]PK11195, can be used to more directly demonstrate the presence of macrophages within the area of inflammation. For infectious diseases, imaging has been used to monitor the pathogen through direct labeling of the pathogen (a more detailed description is below) and assessment of the host response through monitoring inflammatory mediators and cellular and vascular responses [33]. For example, general markers of inflammation like [^18^F]FDG, more selective cellular markers involving direct labeling of neutrophils with a copper-64- or Tc-99m-labeled peptides which bind to the formyl peptide receptor [35–37] or direct labeling of monocyte/macrophages with In-111, R-[^11^C]PK11195, or tracers of matrix degradation, *i.e.*, matrix metalloproteinases (MMPs), and tools for assessment of host responses involved in transcriptional regulation, *i.e.*, NF-κB, and apoptosis, *e.g.*, Tc-99m-labeled annexin V, have been used [38]. The combination of imaging methodologies provides a more thorough understanding of the molecular, cellular, tissue, and organ response to a pathogen or insult that result in an inflammatory response.

Individual and multi-modality imaging approaches can be used to better characterize infections caused by biothreat agents, assess the time course of infection and host response, and evaluate drug distribution, drug targeting, and drug efficacy. Table 2 summarizes the types of physiologic responses and imaging methods used to evaluate the pathophysiology of disease in numerous therapeutic areas and in drug discovery and development that are equally amenable for studying infectious agents. Development of new reporter constructs containing receptor or enzyme reporters in BSL3/4 agents would be beneficial to characterize the distribution and time course of infection within both rodent and non-rodent models using PET and SPECT imaging. Direct labeling of known and novel drugs with PET and SPECT radiotracers serves not only to demonstrate drug biodistribution but also to link plasma and tissue drug levels with pathogen load. Measures of tissue function, metabolism, and activation, *i.e.*, [^18^F]FDG-PET, magnetic resonance spectroscopy (MRS), ASL-MRI, and BOLD-MRI, cell markers, *i.e.*, [^18^F]FEDAC and inflammation, changes in changes in cell phenotype and apoptosis, *i.e.*, Tc-99m-labeled annexin V, and measures of organ/tissue structure *i.e.*, MRI, CT, and ultrasound, all provide tools to dynamically evaluate the host response to the BSL3/4 agent.

**Table 2:** Applications of imaging for the assessment of disease pathophysiology and drug intervention

	Imaging modality					
	Optical	PET	SPECT	CT	Ultrasound	MRI
Physiologic process						
Gene expression	Luciferase-luciferin Green fluorescent protein (GFP) luxABCDE operon Red-shifted firefly luciferase (FFlucRT)	[^124^I/^18^F]FIAU [^18^F]FHBG [^18^F]FMAU [^18^F]fluromethyl-spiperone ^124^I [^18^F]penciclovir	[^125^I]FIAU meta-[^123^I]iodobenzyl-guanidine ^123,131^I			Fe-transferrin receptor
Target expression	Fluorescent dye-conjugated maltodextrin	[^18^F}, [^11^C], [^64^Cu], [^89^Zr]-labeled molecules	[^111^In], [^123^I], [^131^I], [^99m^Tc]-labeled molecules			Chemical exchange saturation transfer (CEST)
Target binding		[^18^F}, [^11^C], [^64^Cu], [^89^Zr]-labeled molecules	[^111^In], [^123^I], [^131^I], [^99m^Tc]-labeled molecules			
Drug PK/PD		[^18^F}, [^11^C], [^64^Cu], [^89^Zr]-labeled molecules	[^111^In], [^123^I], [^131^I], [^99m^Tc]-labeled molecules			
Tissue function						
Metabolism		[^18^F]FDG, [^11^C]lysine [^11^C]palmitic acid, [^11^C]leucine, [^11^C]methionine [^11^C]tyrosine, [^11^C]deprenyl, [^18^F]deoxyuracil				MRS-glutamine, glutamate, choline, creatinine ^13^C-, ^31^P-MRS
Proliferation		[^18^F]FLT				
Apoptosis		[^18^F]-labeled annexin V [^18^F]ML10 [^18^F]ICMT-11 [^18^F]CP18	^99m^Tc-labeled annexin V			
Hypoxia		[^64^Cu]ATSM				
Blood flow		[^15^O]water		Iodine contrast agents	Microbubbles	Arterial spin labeling MRI (ASL) Blood oxygen level-dependent MRI (BOLD) Dynamic contrast-enhanced MRI (dceMRI)
Cell markers						
Inflammatory	GFP-transgenic mice	R-[^11^C]PK11195 [^18^F]FEAnGA [^18^F]FEDAC	^64^CU or ^99m^Tc-labeled peptides [^125^I]DPA173			^19^ F-MRS ^19^ F perfluorocarbon MRS
Neurons		[^18^F]fluorodopa [^11^C]ephedrine				MRS–choline Diffusion tensor imaging (dti-MRI)
Glial cells		R-[^11^C]PK11195				MRS–myoinositol
Organ structure				Bone and lung imaging Soft tissue imaging with contrast agents	Soft tissue	Proton MRI

Based on a review of the literature, imaging has demonstrable advantages over classical methods used for the discovery and development of drugs. Besides reducing the number of animals used in a study, *in vivo* imaging allows one to perform whole body scans, dynamically, with higher statistical power given that each animal can act as its own control and with greater flexibility than classical methods such as histology. Histology is a sensitive measure of disease pathology at a single time point. When imaging is coupled with histology, imaging can be used to dynamically measure disease progression and select cohorts of animals with a similar state of disease for histologic assessment or drug intervention. Imaging approaches as noted in this section have been successfully utilized to characterize a compound’s mechanism of action; to provide a proof of concept or mechanism that a new drug entity engages their respective target; to demonstrate a pharmacologic effect that can provide patient benefit; or to define a drug dose range which is linked to target engagement and where side effects are limited or mitigated. Similar types of questions also exist in the development of countermeasures against high consequence pathogens such as biothreat agents, and application of imaging approaches can provide confidence in the animal model, drug mechanism/target, or drug pharmacodynamics and aid drug development.

## Imaging Applications for Viruses and Bacteria

Imaging applications can be divided into the evaluation of the pathogen specifically and the host response to the pathogen. While all imaging modalities have the potential to assess the pathogen directly, optical and nuclear imaging are typically used because of their inherent sensitivity. Nuclear, MRI, and CT imaging are well suited for assessment of the host response to the pathogen, *e.g.*, morphological changes, organ metabolism, inflammation, and hemodynamics. In the subsequent sections, each approach will be reviewed and divided on the basis of pathogen and host approaches where applicable.

### Viral and Bacterial Reporter Constructs

Viruses engineered with reporter constructs such as enzymes and receptors are amenable to detection by imaging and used to evaluate the biodistribution of viruses containing specific therapeutic or gene transfer payloads. Similarly engineered BSL3/4 viruses can be used to study the virus itself. Numerous reporter constructs have been developed that are detectable by optical, PET, or SPECT imaging. The most often used reporter is herpes simplex virus thymidine kinase (HSV-tk1) with either [^124^I/^18^F]-29-fluoro-29-deoxy-1-b-d-arabinofuranosyl-5-iodouracil ([^124^I/^18^F]FIAU) or 9-(4-[^18^F]fluoro-3-(hydroxymethyl)butyl]guanine ([^18^F]-FHBG) as the enzyme substrate and PET as the imaging modality [39–52]. The viral thymidine kinase is translated in the cell along with the viral RNA and phosphorylates the exogenously delivered radiolabeled [^124^I/^18^F]FIAU or [^18^F]FHBG substrate, trapping it in cells and thereby labeling cells which have been infected with the virus. Alternative reporter enzymes such as human mitochondrial thymidine kinase 2 (hmtk2) [43] and human deoxycytidine kinase (hdCK) [43] utilizing 2′-deoxy-2-[^18^F]fluoro-5-methyl-1-β-l-arabinofuranosyluracil ([^18^F]-FMAU) and 2′-deoxy-2′-[^18^F]fluoroarabinofuranosylcytosine as substrates, respectively, have been developed to avoid immunologic reactions in humans. The varicella zoster virus thymidine kinase (VZV-tk) in combination with radiolabeled bicyclic nucleoside analogs as enzyme substrates is currently being evaluated as a potential reporter construct [45].

Several receptor-based reporter systems such as the rat/human sodium iodide symporter (NIS) [46–48], human norepinephrine transporter (hNET) [49], human somatostatin receptor (hSSTR2) [50], and rat dopamine D2 receptor (D2R) [51] have been incorporated into viruses. Radiotracers amenable for PET or SPECT are available to quantify expression of the different receptors following viral infection. The NIS reporter system is attractive because I-123, I-124, I-131, and Tc-99m pertechnetate can be used which obviates the need for complex chemistry [51]. The hNET reporter system has the advantage of being easily incorporated into the virus because of the small size of the gene cassette [52]. The radiotracers used for detection of hNET are iodine-123, iodine-131, and meta-[^123/124^I]iodobenzyl-guanidine. While hSSTR2 tracers such as [^68^Ga]DOTA-TOC, [^68^Ga]DOTATATE, and [^111^In]DOTABASS provide high specific binding and low background due to their rapid clearance, the fact that DOTATOC and DOTATATE are agonists for the G protein-coupled receptor, they can perturb cellular function and confound the interpretation of the results. Incorporation of the D2R into a virus and imaging receptor expression with [^18^F]-fluromethyl-spiperone has been utilized; however, the use of a mutant (D2R80A) proved more valuable because it completely uncoupled ligand binding with activation of G protein-linked signaling and adverse effects on the transduced cells [51]. Similar receptor-based systems can be constructed with an MRI detectable reporter, *e.g.*, transferrin receptor and iron accumulation [53, 54]. Despite significant spatial resolution, such MRI-based approaches lack the sensitivity of nuclear and optical approaches and present potential artifacts due to the longevity of the signal that may be associated with remnants of cells containing the iron particles. With regard to creating reporter systems in bacteria, less work has been done.

While reporter systems have been developed to track therapeutic genes associated with gene therapy, or stem cells to assess viability and longevity or to act as radiolabeled suicide molecules for oncolytic therapy, the procedures developed for insertion of such reporters and evaluation of their functionality can also be applied to studying the virus or bacterial particle itself. Optical imaging and viruses/bacteria containing reporter constructs such as luciferase/luciferin or green fluorescent protein which are amenable to optical imaging have been utilized to evaluate pathogens and are reviewed in the next section.

### Optical Imaging

Bioluminescence approaches have been utilized to characterize the biodistribution of viral and bacterial pathogens post-infection. Luciferase which requires exogenous luciferin administration or the lux operon containing both luciferase and luciferin has been transfected into murid herpesvirus-4 [55], varicella zoster [56], Venezuelan equine encephalitis [57], Chikungunya [58], cowpox [59], and murine cytomegalovirus [60] viruses and bacteria such as *Staphylococcus aureus* [61], enteropathogenic *Escherichia coli*, and enterohemorrhagic *E. coli* [62], *Bacillus anthracis* [63], *Yersinia pestis* [64–66], *Francisella tularensis* [67], and *Burkholderia mallei* and *pseudomallei* [68, 69]. Incorporation of the optical reporter in the virus/bacteria noted above was shown to not markedly affect growth rate, survival, and infectivity and functioned similarly to the wild-type pathogen. Factors influencing the expression of the bioreporter were the choice of promoter, size of the amino acid flanking regions, and whether the construct was inserted randomly or site-specifically. The bioreporters allowed for whole body detection of pathogens, and assessment of the time course of infection and the bioluminescent signal was highly sensitive with the signal intensity correlating to pathogen number.

Site-directed insertion of the luciferase construct is important in preserving viral function, and when coupled with the constitutive native promoters for specific viral proteins, optical imaging can be used to assess early and late stage viral replication. Insertion of the luciferase expression cassette in murid herpesvirus-4 between open reading frames open reading frames (ORF) 57 and 58 which are involved in lytic replication and viral spread allowed for the monitoring of virus distribution and function by bioluminescence [55]. The addition of the bioreporter was shown to not markedly affect viral functions. In varicella zoster virus, placement of click beetle luciferase in-frame with ORFs 63, 68, and 70 and under their constitutive promoters not only allowed for optical imaging of the virus but also monitoring of IE63 and gE luciferase fusion proteins which are involved in early and late varicella replication, respectively [56]. These two studies demonstrate that placement of the reporter constructs can provide more than just virus location but also information about the functioning of the virus and the role of specific viral proteins in replication and intercellular infection.

Viral luciferase expression in combination with whole body optical imaging has been used to characterize different routes of viral infection and drug efficacy. Intranasal infection with murid herpesvirus-4 resulted in nasal and lung expression of luciferase activity and abdominal organ expression following intraperitoneal infection, while no signal was detected following oral administration [55]. Intranasal infection with VEEV containing a luciferase bioreporter demonstrated brain uptake of the virus 3 days prior to clinical signs [57]. Chikungunya virus with a luciferase reporter injected into the mouse footpad resulted in local bioluminescence but not systemic signal [58]. Treatment of varicella virus-infected mice with valacyclovir [56] and VEEV-infected mice with Ampligen, a TLR-3 agonist [57], resulted in a decrease in luciferase luminescence which is suggestive of a reduction in viral replication. It is interesting to note that optical imaging was also able to detect upon withdrawal of valacyclovir that the luminescence signal reappeared, reflecting renewed viral replication. Cidofovir treatment at doses of 25 and 100 mg/kg administered intraperitoneal (i.p.) to mice infected with a green fluorescent protein (GFP)-expressing cowpox virus significantly reduced the bioluminescent signal which is indicative of reduced viral replication when the animals were imaged post-mortem [59]. In mice infected with a GFP-expressing mouse cytomegalovirus (GFP-MCMV), i.p. administration of 100 mg/kg and subcutaneous (s.c.) administration of 50 mg/kg ganciclovir decreased the fluorescent signal [60]. Treatment of GFP-MCMV mice with 50 mg/kg, s.c., of a novel compound identified by screening, 1-(3,5-dichloro-4-pyridyl) piperidine-4-carboxamide (DPPC), delayed the appearance of a muted fluorescent signal [60]. These data taken together indicate that optical imaging is a sensitive tool capable of characterizing virus distribution, the effect of drug intervention and discerning differences between dose and route of drug administration.

Dynamic, real-time optical imaging of bacteria transfected with various bioluminescent reporters has exposed pathways of infection which were previously undetected. Construction of *B. anthracis* containing the lux operon established that following inhalation and cutaneous infections, the *B. anthracis* spores germinated at the site of inoculation while Peyer’s patches were the main site of bacterial growth following intragastric inoculation [63]. Contrary to our current understanding, optical imaging established that *B. anthracis* germination occurred at the site of inoculation and initially did not require transport to the draining lymph nodes for propagation, but eventually all routes of infection progressed to the lymph nodes. Following intranasal *B. mallei* infection, a strong luminescent signal was observed in the lung that could be reduced by intraperitoneal administration of levofloxacin, 24 h post-infection [68]. Similarly, intranasal *B. pseudomallei* exhibited a similar pattern of infection but in addition optical imaging demonstrated that the olfactory nerve was the route of entry for the bacteria into the brain, and such entry occurred prior to bacteria being detected in the blood [69]. *Y. pestis* which is a gram-negative bacterium capable of causing bubonic, septicemic, and pneumonic plague has been evaluated utilizing optical imaging approaches to track both level and distribution of infection and the effect of pharmacologic intervention [64–66]. Insertion of the luxCDABE operon driven by either the PtolC or PcysZK promoter into *Y. pestis* provided a tool to evaluate differing routes of administration, *i.e.*, intradermal, subcutaneous, or intranasal. With the intradermal and subcutaneous routes, bioluminescence from the LuxPcysZK strain was detectable in the draining lymph nodes followed by systemic dissemination while intranasal exposure localized the signal to the lungs and thoracic cavity [64]. Utilization of a non-disseminating LuxPcysZK ∆pla construct demonstrated that the bioluminescent signal was confined to the site of inoculation as expected. Some investigators [65] have also shown that bioluminescence signal from *Y. pestis* CO92 pLux is capable of detecting 10^4^−10^5^ cfu and is linearly correlated with the degree of infection. Unlike excising the organs and counting colonies, the optical imaging approach monitored the fairly variable progression of infection in each animal, staging the degree of infection for treatment group selection and thereby reducing the variability in the measurement. Levofloxacin treatment (10 mg/kg/day for 6 days, 24 h after infection) significantly reduced the luminescent signal indicating that the treatment killed the bacteria [66]. Thus, for *B. anthracis*, *B. mallei*, *B. pseudomallei*, and *Y. pestis*, optical imaging was a powerful tool to track the course of infection, define new pathways of infection, and sensitive enough to discern changes due to drug treatment.

Beyond using optical imaging to assess the degree and localization of bacterial infections, it can also be coupled with other optical reporters to better understand the pathophysiology of the disease and consequences of drug intervention. For example, in a skin wound model, a bioluminescent *S. aureus* strain (SH1000) was used to track the course of wound infection [61]. Performing the same experiment in LysEGFP mice, which possesses green fluorescent neutrophils, one was able to assess both bacterial infection and degree of inflammation as assessed by neutrophil infiltration using bioluminescence and fluorescent optical imaging. Optical imaging has also proved viable for the assessment of vancomycin–rifampin efficacy against *S. aureus*-induced prosthetic joint infection in mice [70].

Improvements in optical reagents are ongoing to both increase the sensitivity of optical imaging and to be able to image deeper organ structures. Incorporation of a red-shifted derivative of firefly luciferase (FFlucRT) into *Mycobacterium tuberculosis* [71] or use of a near-infrared fluorogenic substrate against the endogenous *M. tuberculosis* β-lactamase [72] increased sensitivity to detect 10^4^ and 10^2^ colony-forming units, respectively. The red-shifted and near-infrared agents also increased the depth from which the optical signal can be detected. Targeting specific bacterial transport pathways or secreted enzymes has also proved viable for use in detecting infection. For example, fluorescent dye-conjugated maltodextrin-based imaging probes are internalized in bacteria expressing the specific transporter and can be used for detection of these bacteria [73]. Probes which are normally silent but fluoresce when activated by secreted nucleases have been used to demonstrate the distribution of *S. aureus* in infected animals [74]. Thus, generation of general and targeted reagents is another mechanism to improve the utility and sensitivity of optical imaging.

Optical imaging while limited to mouse models of infection has established the utility of imaging to better understand viral and bacterial infections, routes of infection, and the effect of drug intervention. Based on what has been learned through optical imaging, one would expect that similar tools could be built for use in nuclear imaging, *i.e.*, PET and SPECT. The methods employed to build the tools for optical imaging can be instructive in building the pathogen constructs amenable to nuclear imaging. The nuclear imaging tools broaden applicability from rodents to primates due to the ability of imaging deeper organs and as such expand the number of animal models within which to evaluate viral/bacterial distribution and pathophysiology. The opportunity to evaluate viral/bacterial pathogens in non-rodent models also provides a mechanism to evaluate drugs for the treatment of BSL3/4 agents in models which might be more predictive of the human condition and aid in the registration of new drug applications under the U.S. FDA Animal Rule.

### Nuclear Imaging

Despite the amount of work performed with optical imaging to develop transduced viruses as tools to monitor gene therapy, oncolytic therapy, and stem cell survival [39–54], a minimal amount of work has been done to develop tools amenable to nuclear imaging to directly characterize viral/bacterial distribution. A few examples utilizing bacteria have been reported. Direct labeling of an attenuated *Salmonella abortusovis* with technetium-99m was performed and evaluated in sheep [75]. Labeling efficiency was low, *i.e.*, 30 %, but bacterial viability was unchanged and the investigators were able to discern the spatial and temporal patterns of bacteria dissemination in the lymphatic system following a sub-cutaneous injection. Several other investigators utilizing Tc-99m or indium-111 (In-111) directly labeled *Pseudomonas aeruginosa*, *E. coli*, *Streptococcus*, and *S. aureus* to perform similar types of experiments [76–81]. While labeling the pathogen directly appears to be a viable approach, only acute evaluation is possible given that with bacterial replication the signal is diluted and the potential for freely circulating radiolabel can confound the results. Several studies utilized the constitutively expressed bacterial thymidine kinase (TK) or transfected TK and the substrate, 1-(29deoxy-29-fluoro-b-Darabinofuranosyl)-5-[^125^I]iodouracil ([^125^I]FIAU), which is amenable to SPECT imaging to monitor bacterial infections. Constitutively expressed bacterial TK in *E. coli*, *E* *nterococcus* *faecalis* 49532, *Staphylococcus pneumonia* 49619, *Staphylococcus aureus* 29213 and 25293, *Staphylococcus epidermidis* F362 were successfully imaged by SPECT using [^125^I]FIAU to demonstrate distribution post-infection [82]. Others have also shown that the [^125^I]FIAU signal is strongly correlated with bacterial *E. coli* load with a limit of detection of 10^9^ colony-forming units/ml [83]. In bacteria not expressing TK, incorporation of bacterial TK into *M. tuberculosis* under the HSP60 promoter generated a tool (*M. tuberculosis* Phsp60 TK) for use in assessing infection associated with tuberculosis [84]. As with the other studies noted above, SPECT imaging utilizing [^125^I]FIAU was used to assess the degree of bacterial infection and localization of the bacteria in the lung.

A few studies employing nuclear imaging approaches have focused on studying the pathophysiology of infection and the distribution of therapeutics designed to treat the infection. Neuroinflammation related to exposure to herpes simplex virus 1 (HSV-1) was assessed by PET by quantifying β-glucuronidase secretion from activated microglia and by direct labeling of activated microglia using a marker for the PBR [85]. To measure β-glucuronidase activity, a PET tracer, 1-O-(4-(2-[^18^F]fluoroethyl-carbamoyloxymethyl)-2-nitrophenyl)-O-β-d-glucopyronuronate ([^18^F]FEAnGA), which acts as a substrate for the enzyme was synthesized. Upon cleavage of [^18^F]FEAnGA by β-glucuronidase, [^18^F]fluoroethylamine is released and because it is slowly cleared from tissue demonstrates areas of increased enzyme activity associated with areas of neuroinflammation. While there was not a one-for-one correspondence between R-[^11^C]PK11195 and [^18^F]FEAnGA as had been expected, the investigators demonstrated that there was a relationship between tracer binding and the symptom score which implies that PET imaging was able to more objectively stage the level of neuroinflammation associated with HSV-1 infection and thereby more accurately select cohorts for comparison [85]. Lung inflammation associated with exposure to the pandemic influenza virus (H1N1pdm) [86] and *M. tuberculosis* [87] was assessed using PET and the glucose analog, [^18^F]FDG. The investigators correlated the increase in glucose metabolism with viral titers. Like with neuroinflammation, direct measures of the 18-kDa translocator protein or PBR have been performed following a lung lipopolysaccharide challenge utilizing another selective ligand, *N*-benzyl-*N*-methyl-2-[7,8-dihydro-7-(2-^18^F]fluoroethyl)-8-oxo-2-phenyl-9H-purin-9-yl]acetamide ([^18^F]FEDAC) [88]. [^18^F]FEDAC binding increased with the severity of lung inflammation and primarily localized to neutrophils and macrophages. A new SPECT tracer, [^125^I]Iodo-*N*,*N*-diethyl-2-]2-(4-methoxy-phenyl)-5,7-dimethyl-pyrazolo[1,5-a]pyrimidin-3-yl]-acetamide ([^125^I]DPA713), has been developed and demonstrated to have higher signal-to-noise ratios than R-[^11^C] PK11195 and lower lipophilicity [89, 90]. Measures of tissue hypoxia utilizing [^64^Cu]Copper-diacetyl-bis(N^4^-methyl-thiosemicarbazone (which is [^64^Cu]ATSM) have demonstrated that *M. tuberculosis*-induced tuberculosis lesions in mice are hypoxic and sensitive to varying drug regimens [91]. While these nuclear imaging approaches do not directly measure the degree of infection, they are informative with regards to characterizing the host response to the virus which can be used to identify treatment and potentially new drug discovery approaches.

Evaluation of radiolabeled drugs as tools for nuclear imaging is a new approach to characterizing infection as well as evaluating the pharmacokinetics of the therapeutic. Isoniazid (INH) is routinely used to treat tuberculosis. Utilization of 2-[^18^F]fluoroisonicotinic acid hydrazide (2-[^18^F]INH) and evaluation in *M. tuberculosis*-infected animals demonstrated that 2-[^18^F]INH accumulates in the lung at sites of infection and becomes associated with the mycobacterium such that the radiolabel can be a direct marker of *M. tuberculosis* [92]. In addition to demonstrating drug exposure and pathogen presence, target-specific compounds amenable to radiolabeling and PET or SPECT imaging can be used to study various processes associated with infection. For example, in a review by Bray and colleagues [93], they hypothesized that radiolabeled compounds can be used to demonstrate that targeted therapeutics bind to the viral envelope glycoprotein, NS3 protease, RNA replication complex, or cell surface E1-E2 protein. Some therapeutic agents exist [93, 94] but little work has been done to radiolabel the molecules and use them as tools to better understand the processes involved in infection and virus/bacteria replication. In addition to small molecules, there are opportunities to radiolabel biologics such as antibodies, mini-bodies, and diabodies as has been demonstrated in the cancer field [95–97]. Based on targeted screening, tools that are more selective for specific viral and bacterial processes could be generated.

### Magnetic Resonance Imaging

A few papers have been published describing the use of MRI in the evaluation of viral and bacterial infections to most notably assess bacterial distribution [97] and germination/proliferation [98]. *S. aureus* was labeled with iron oxide nanoparticles which remained on the bacterial surface [97]. The labeling of the bacteria had no effect on growth and ability to infect human umbilical vein endothelial cells *in vitro* [97]. Upon infection, MRI was able to detect *S. aureus* up to five cycles of cell division and with a minimum detection limit of 10^5^ bacteria colony-forming units. In addition, upon macrophage phagocytosis of the labeled bacteria, MRI detected the resulting inflammation associated with infection. Chemical exchange saturation transfer (CEST) MRI, albeit challenging to implement, has recently been used to show that endogenous bacterial contrast can be used to monitor the germination and proliferation of bacteria [98].

Magnetic resonance imaging has also proved useful in evaluating the host response to infection by measuring inflammation [99], brain neurochemical changes [100], and lung pathology [101]. Investigators have employed fluorine-19 (F-19) perfluorocarbon emulsions as a means of assessing macrophage infiltration following *S. aureus* infection since the macrophage phagocytosis the F-19 emulsions and F-19 can be detected by MRS [99]. The ^19^F MRS signal was detected as early as 48 h post-infection and out to 9 days post-infection [102]. Mice infected with *S. aureus* Xen29 containing the optical reporter luxABCDE operon induced a thigh abscess, and following 30 mg/kg/day vancomycin or 15 mg/kg/day of linezolid for 7 days, a reduction in both F-19 signal by MRS and bioluminescent signal was observed indicating both a decrease in inflammation and infection, respectively [102]. MRS has been applied to simian immunodeficiency virus (SIM)-infected rhesus macaques to study the acute effects of virus on brain neurochemistry, reflective of changes in neuronal health [100]. While the changes noted were brain region specific, focusing on the frontal cortex reductions in *N*-acetyl aspartate, a measure of neurodegeneration, increases in choline and myoinositol, measures of gliosis, and no change in creatinine and glutamate/glutamine were noted at 2 weeks post-infection that resolved by 4 weeks [100]. The changes were highly correlated with the level of viremia and demonstrated that MRS is capable of detecting subtle changes consistent with neurodegeneration/neuroinflammation post-SIM infection. ^1^H-MRI and ^3^He diffusion MRI have proven to be useful tools in evaluating lung architecture following Sendai virus infection [101]. Given that ^3^He does not diffuse across the alveolar wall, it is very sensitive to defining and quantifying small microstructural changes and combined with ^1^H MRI establishing that airspace enlargement developed following virus infection [101].

*In vivo* imaging taken in its entirety has proven to be a sensitive tool for monitoring the distribution of engineered viruses and bacteria by either optical or nuclear imaging approaches and for assessing the consequences of infection. In addition to characterizing disease progression, imaging is a sensitive tool for assessing the degree of viral and bacterial infections relative to plasma titers and for monitoring the effects of drugs on pathogen growth and progression of disease. Despite the amount of work done in the area of optical imaging, opportunities exist to employ nuclear and MRI imaging approaches to perform similar types of studies in non-rodent models and also apply the tools to the development of new drugs by correlating drug distribution and levels with pathogen expression, by linking drug exposure with pharmacologic effect and by establishing predictive preclinical models of drug efficacy that can serve as a surrogate for phase II/III clinical studies given the issues with development of countermeasures for BSL3/4 biothreat agents.

## Conclusions

The pathogenesis of viral and bacterial infections involves pathogen-specific activities such as binding, internalization, replication, muted host anti-viral or anti-bacterial response, and budding and host-specific responses that result in disease. Drug discovery efforts could focus on eliminating the pathogen or on treating or preventing the resulting disease, of which the latter could also have broader application in improving human health. *In vivo* imaging can be directly applied to better understand the above processes associated with the natural history of infectious agents, the host response, and the discovery and development of drugs for the treatment of infections caused by BSL3 and BSL4 threat agents.

Classical approaches to the study of BSL3/4 infections involve serial necropsies, tissue dissection, plaque counts, and immunohistochemistry to demonstrate the distribution of pathogens and degree of infection, drug extraction from tissues as a measure of drug exposure, and mortality as endpoints for assessing drug efficacy. However, numerous other questions requiring a dynamic assessment of infection and pathophysiology remain unanswered. In addition to knowing that an animal has been exposed to an aerosolized dose of a pathogen, one needs to determine the dose of pathogen received within the lung and the relationship of breathing kinetics to dose. Since distribution of the pathogen in the tissues and organs may differ acutely post-infection *versus* late in infection, it is important to map the differences so as to insure proper drug exposure late in infection. While the degree of viremia or bacteremia and plasma drug levels may be good measures to demonstrate infection and the potential for drug efficacy, respectively, correlating measures of tissue pathogen and tissue drug concentrations may be more important to link efficacy with drug dose. In some instances like with VEEV, one must know both the time course and route of brain infection and whether the therapeutic agent under development reaches the brain in sufficient quantities to result in a beneficial effect. In all cases noted above, dynamic real-time measurements can provide important data to track infection, host response, and time course for intervention and to assess drug efficacy rather than rely on mortality as the sole endpoint. By using dynamic measures of drug efficacy, one might be better able to discern potential mechanisms for efficacy and potential targets/target organs to refine the drug discovery approach and/or compound.

In summary, *in vivo* imaging can provide real-time, in-life measures of (1) target distribution and drug exposure, (2) binding of drug to the target, and (3) physiologic or pharmacologic consequences of pathogen or drug intervention. Taken together, *in vivo* imaging can be used to evaluate countermeasures against BSL3/4 threat agents to answer these three fundamental questions and to develop countermeasures under the U.S. FDA Animal Rule provision with higher confidence of clinical success and benefit.

## Abbreviations

[^18^F]FDG: 2-Deoxy-2-[^18^F]fluoro-d-glucose
[^18^F]FLT: [^18^F]fluoro-3-dexoythymidine
[^18^F]PF-05270430: 4-(3-[^18^F]fluoroazetidin-1-yl)-7-methyl-5-{1-methyl-5-[4-(trifluoromethyl)phenyl]-1H-pyrazol-4-yl}imidazo[5,1-f]-[1,2,4]triazine
[^124,125^I/^18^F]FIAU: [^124,125^I/^18^F]-29-fluoro-29-deoxy-1-b-d-arabinofuranosyl-5-iodouracil
BSL: Biosafety level
CHIKV: Chikungunya virus
CT: Computed tomography
EEEV: Eastern equine encephalitis virus
MRI: Magnetic resonance imaging
MRS: Magnetic resonance spectroscopy
PET: Positron emission tomography
RRV: Ross River virus
SINV: Sindbis virus
SPECT: Single photon emission tomography
US: Ultrasound
VEEV: Venezuelan equine encephalitis virus
WEEV: Western equine encephalitis virus
